# Adiponectin in relation to exercise and physical performance in patients with type 2 diabetes and coronary artery disease

**DOI:** 10.1080/21623945.2021.1996699

**Published:** 2021-11-14

**Authors:** Hani Zaidi, Rune Byrkjeland, Ida U. Njerve, Sissel Åkra, Svein Solheim, Harald Arnesen, Ingebjørg Seljeflot, Trine B. Opstad

**Affiliations:** a Center for Clinical Heart Research, Department of Cardiology, Oslo University Hospital Ullevål, Norway; b Faculty of Medicine, University of Oslo, Oslo Norway

**Keywords:** Adiponectin, vo_2_peak, exercise-training, coronary artery disease, type 2 diabetes

## Abstract

Introduction: Adipokines, expressed by adipose tissue (AT), have been associated with metabolic disturbances and coronary artery disease (CAD). The impact of exercise training on the AT in patients suffering from both diabetes and CAD is unknown. To gain knowledge on changes in ATs’ inflammatory profile in such a population, we investigated the effects of long-term exercise on selected adipokines and their associations with physical performance and glucometabolic variables. Adiponectin was selected based on its anti-atherogenic and anti-diabetic properties and visfatin and tumour necrosis factor (TNF) for their association with atherosclerosis and metabolic disorders. Not many studies have focused on the effects of long-term exercise training on adipokines in patients with concomitant T2DM and CAD. Methods: Patients with type 2 diabetes and CAD (n = 137), 41–81 years, 17.2% females, were randomized in a 1:1 manner to an exercise group, who underwent 1 year of 150 min weekly combined strength and endurance exercise, or a control group. AT from the gluteal region and blood samples were obtained at baseline and after 12 months, along with a physical performance test, assessed by the VO_2_ peak. Circulating protein levels were measured by ELISA. RNA was extracted from AT and expression levels were relatively quantified by PCR. Results: After 1 year, no significant difference in the change in the investigated markers between the intervention group and the control group was observed. Changes in circulating adiponectin and VO_2_ peak correlated in the total population (r = 0.256, p = 0.008). At baseline, circulating adiponectin and TNF correlated inversely with insulin and with C-peptide and VO_2_peak, respectively (p < 0.001, all). Conclusion: In this population with concomitant diabetes and CAD, ATs’ inflammatory profile remained unchanged apparently after 1 year of exercise intervention. Changes in the VO_2_peak were nevertheless, related to changes in circulating adiponectin levels. Trial registration: http://www.clinicaltrials.gov NCT01232608.

## Introduction

Adiponectin is an adipocyte-derived protein that plays a major role in metabolic disorders such as type 2 diabetes (T2DM), insulin sensitivity, metabolic syndrome (MetS) and coronary heart disease. Although mainly produced by the adipose tissue (AT), several reports have shown that scarce amounts of adiponectin are produced by different tissues and cells, such as cardiomyocytes, skeletal muscle, osteoblasts and the liver[1]. With its anti-diabetic and anti-atherogenic properties, adiponectin has been shown to be inversely associated with body fat mass and other cardiovascular disease (CVD) risk factors [2–4]. Low levels of circulating adiponectin have also been associated with insulin resistance, dyslipidemia and atherosclerosis[5]. By inducing expression of peroxisome proliferative-activated receptor (PPAR)-α and inhibiting tumour necrosis factor (TNF), adiponectin is able to increase insulin sensitivity and mediate its anti-inflammatory effects [5–7].

Altered properties of the AT with a subsequent reduction in adiponectin and increased TNF secretion may thus result in an imbalance of the glucose homoeostasis, precipitating metabolic disorders. Circulating TNF, shown to be increased in CAD patients[8], seems to correlate with insulin resistance and pancreatic β-cell function in T2DM patients[9]. TNF modulates the insulin receptor through serine phosphorylation and subsequent tyrosine kinase inhibition [10] and also inhibits glucose-induced insulin released from pancreatic beta cells through the activation of nuclear factor kappa B [11,12].

Contrary to TNF, visfatin seems to bind and sensitize the insulin receptor, reducing glucose release from hepatic cells and increasing glucose consumption in adipocytes, thereby causing decreased circulating glucose levels[13]. AT expression and circulating visfatin levels seem to increase in parallel with obesity[14]. In patients with T2DM, elevated visfatin levels were associated with atherosclerotic disease, proposed to serve as a potential predictor of atherosclerotic plaques [15,16]. Visfatin was also shown to induce proliferation and inflammation in endothelial and smooth muscle cells, qualifying it as a potential marker for endothelial dysfunction[17]. Visfatin is mainly expressed in the visceral AT, although identified in several tissues including the brain, spleen, kidneys and lungs. Contrary to visfatin, adiponectin is decreased in patients with T2DM and inversely related to traditional cardiovascular risk factors[18]. Hence, despite the fact that both adiponectin and visfatin have insulin-mimetic effects, their association with obesity, insulin resistance and cardiovascular disease seems to go in opposite directions, thus of particular interest to study. Additionally, the combination of low adiponectin and high serum visfatin seems to be consistently associated with an unfavourable metabolic profile [19–21].

TNF has been reported to be strongly involved in the regulation of both adiponectin and visfatin, as shown by the interesting study of Hector et.al[22].

Physical activity is effective in primary prevention of CVD, partly by regulating glucose metabolism and increasing the action of insulin [23,24]. Although limited data exist on the effects of exercise on AT-expressed adipokines, excercise combined with diet-induced weight loss seemed to increase circulating levels of adiponectin in obese and insulin-resistant patients [25,26] and multiple studies have confirmed the effects of exercise on reducing serum visfatin concentration, although contrary observations have also been made[27]. Whilst there are no robust and unanimous results, due to variations in modality, intensity and duration of the exercise intervention across different studies[28], review articles indicate that a balanced, low-in-fat diet along with targeted physical exercise may influence serum visfatin levels[27].

To obtain more insight into the connection between the serum levels and AT-expressed adipokines with metabolic traits, exercise and physical fitness, we investigated effects of one-year exercise training on adiponectin, visfatin and TNF in patients with concomitant CAD and T2DM[29]. Our hypothesis was that gene expression and serum levels of the selected adipokines would change in a beneficial manner, namely, an increase in adiponectin levels and a decrease of visfatin and TNF. In parallel, associations between the investigated markers, glucometabolic variables and physical performance assessed by the VO_2_ peak were further explored.

## Materials and methods

### Study population

The present investigation is a sub-study of the EXCADI (exercise training in patients with coronary artery disease and type 2 diabetes) trial, in which patients with T2DM and CAD were included at Department of Cardiology, Oslo University Hospital – Ullevaal, between August 2010 and March 2012. The study design has been described previously[29]. In brief, 137 participants with known T2DM and stable CAD were randomized in a 1:1 manner to an exercise group or a control group. The exercise group was assigned to a 12-month exercise programconsisting of a 150 min weekly combined strength and endurance exercises, whereas the General Practitioner followed up the control group in a conventional manner. Exclusion criteria were the presence of proliferative retinopathy, end-stage renal disease, cancer, stroke or acute myocardial infarction (MI) within the last 3 months, unstable angina, decompensated heart failure, serious arrhythmia, severe valvular disease, severe rheumatologic disease, chronic obstructive pulmonary disease stadium GOLD IV, thromboembolic disease, ongoing infections, severe musculoskeletal disorders and other disabilities limiting the ability for physical activity.

A cardiopulmonary exercise test (CPET) was performed at the beginning and at the end of the study. A VO_2_ peak was defined as the highest consecutive 30s average of oxygen uptake during the test, measured by breathing through a two-way breathing mask. In the main EXCADI study, we could not demonstrate effects of exercise intervention on the VO_2_ peak or HbA1c. However, these parameters did improve in the subgroup of patients without advanced vascular disease[29].

The EXCADI study was conducted in accordance with the Declaration of Helsinki. It was approved by the Hospitals Data Protection Services and the Regional Committee of Medical Research Ethics in South-Eastern Norway (2010/1060) and all patients gave their written informed consent to participate.

### Physical exercise intervention

The physical exercise program was designed in collaboration with the Norwegian School of Sport Sciences. The details of the program have been described previously[29]. Briefly, the exercise group had two group-based sessions with a variety of strength and endurance exercises and a home-based session, which amounted to a total of 150 min of exercise per week. Approximately two thirds of the exercise was aerobic and one third was resistance training.

### Laboratory methods

Venous blood samples were collected by standard venipuncture between 08.00 am and 10.00 am in fasting condition, before intake of morning medication both at inclusion and after 12 months. AT samples were taken from the gluteal region and snap frozen immediately at −80°C until extraction of RNA. Blood samples, including HbA1c, insulin and C-peptide, were acquired and measured through conventional routine methods. Serum was prepared for establishing a biobank by centrifugation within 1 hour for 10 min at 2500 x g. Determination of circulating adiponectin, visfatin and TNF was performed by the following ELISA methods: Human total Adiponectin/Acrp30 (Catalogue # DRP300) and Human TNF-α (Catalogue # HSTA00E) (both R&D Systems and Human Visfatin) (Catalogue # MBS723926) (MyBioSource). The inter-assay coefficients of variation in our laboratory were 8.0%, 4.2% and 8.1%, respectively.

Total RNA from AT was isolated, including disruption and homogenization in Tissue lyser (Qiagen), by the use of a High Pure RNA Tissue Kit (Catalogue #12,033 674,001) (Hoffman-La Roche Ltd.), according to a combination of the kit protocol and previous experience in our laboratory. Minor modification included adding Proteinase K after the homogenization procedure for the inactivation of DNAses and RNAses. RNA quality and quantity (ng/µL) were determined using a NanoDrop™ 1000 Spectrophotometer (Nanodrop Technologies). Extracted RNA was stored at −80°C until analyses. Copy DNA (cDNA) was synthesized from an equal amount of RNA (5 ng/ųl) with qScript™ cDNA superMix (Catalogue # 95,048–100) (Quanta Biosciences Inc.). Real-time PCR was performed with TaqMan Low Density Custom Arrays on a ViiA™7 instrument, using TaqMan® Universal PCR Maser Mix (P/N 4324018) and TaqMan® assays for adiponectin (Hs00605917_m1), visfatin (Hs00237184_m1) and TNF (Hs01113624_g1) (Applied Biosystems, by Life Technologies). β-2-microglobulin (Hs99999907_m1) (Applied Biosystems) was used as the endogenous control, and mRNA levels were determined by relative quantification (RQ) using the ΔΔCT method[30].

### Statistics

The demographic data are given as proportions, mean (±standard deviation) for data with equal distribution or as median (25^th^ and 75^th^ percentile) for skewed data. The Mann-Whitney U test or Student’s t-test was used for analysis of continuous variables and Chi-square test for categorical variables as appropriate for differences between groups. The Wilcoxon Signed Rank test was used for analysis of within-group changes after intervention. Differences in changes during the intervention period (delta) between the randomized groups were analysed by the Mann- Whitney U test. Correlations analyses were analysed by Spearman’s rho and adjusted for by Bonferroni correction. Power calculations were performed in the main study based on expected HbA1c reductions, thus not for considering effects on the investigated adipokines. Statistical calculations were performed using SPSS version 26 (SPSS Inc., Chicago, Illinois, USA). P-values *<0.05* were defined as statistically significant.

## Results

Baseline characteristics of the total population are shown in Table 1. Of the 137 included patients, 2 dropped out after randomization, 5 could not be contacted, 5 dropped out due to medical reasons, 2 were dissatisfied with randomization to the control group and 9 were excluded from the study due to low adherence to the intervention protocol. Blood samples from 2 patients were missing and thus, 112 patients were analysed for the intervention effect, which includes compliant participants with available blood samples (51 and 61 in the exercise and control groups, respectively). No significant differences in baseline characteristics between the randomized groups were observed (Table 1).

**Table 1.** Baseline characteristics of the total study population and according to the randomized groups in those completing the study (n = 112)

	All (137)	Exercise (51)	Control (61)	P-value
Age	63 (± 8)	65 (± 8)	63 (±7)	*0.376*
Sex (m/f)	115/22	45/7	51/11	*0.422*
Previous AMI, n (%)	62 (45)	20 (39)	31 (49)	*0.248*
Advanced vascular disease, n (%)^a^	79 (57)	28 (54)	41 (66)	*0.221*
CHF, n (%)	11 (8)	2 (4)	5 (8)	*0.361*
PAD, n (%)	13 (9,5)	3 (6)	6 (10)	*0.456*
Years with T2DM	9.0 (5.0,15.0)	11.0 (5.0,15.0)	9.0 (5.5,13.5)	*0.322*
Hypertension, n (%)	100 (73)	39 (75)	48 (76)	*0.882*
Current smokers, n (%)	23 (16.8)	9 (17)	9 (14)	*0.657*
SBP (mmHg)	138 (127,150)	136 (129,150)	140 (126,150)	*0.800*
DBP (mmHg)	79 (71,86)	76 (71,82)	81 (71,87)	*0.080*
Weight (kg)	86.5 (77.1,97.0)	87.0 (78.5,99.0)	85.0 (77.5,96.0)	*0.732*
HbA1c (%)	7.4 (± 1.34)	7.4 (±1.4)	7.4 (± 1.1)	*0.751*
Insulin (pmol/L)	57 (33, 101)	54 (31,95)	63 (32,104)	*0.432*
C-peptide (pmol/L)	965 (713,1290)	956 (637,1165)	1042 (743,1453)	*0.089*
Total cholesterol (mmol/L)	3.9 (±0,96)	3.8 (±0.82)	4.2 (±1.07)	*0.229*
Triglycerides (mmol/L)	1.42 (1.06,1.91)	1.44 (1.09,1.86)	1.36 (0.99,1.88)	*0.555*
LDL (mmol/L) cholesterol	2.0 (1.6,2.6)	1.8 (1.5,2.5)	2.2 (1.6,2.9)	*0.153*
HOMA2-IR	1.3 (0.7,2.1)	1.1 (0.7,1.9)	1.3 (0.7,2.2)	*0.577*
BMI (kg/m^2^)	28.7 (25.7,31.6)	29.4 (25.5,31.8)	28.1 (25.6,31.6)	*0.675*
**Medication, n (%)**				
ACE inhibitors	43 (31.6)	14 (27)	21 (34)	*0.423*
A2 blockers	55 (40.1)	20 (38)	25 (40)	*0.894*
Statins	128 (93.4)	49 (94)	59 (95)	*0.897*
Metformin	101 (73.7)	40 (77)	46 (74)	*0.631*
Sulfonylureas	48 (35.0)	23 (44)	18 (29)	*0.081*
Gliptin	17 (12.4)	6 (12)	11 (18)	*0.373*
Insulin	26 (19.1)	12 (17.4)	14 (20.9)	*0.603*
Anti-platelet drugs	129 (94)	47 (90)	61 (98)	*0.054*

Values are given as number (proportions), mean (±SD) or median (25 and 75 percentiles) AMI; acute myocardial infarction, SBP; systolic blood pressure, DBP; diastolic blood pressure, T2DM; type 2 diabetes mellitus, HbA1c; glycylated haemoglobin, LDL; low-density lipoprotein, CHF; congestive heart failure, PAD; peripheral artery disease, HOMA2-IR; homoeostatic model assessment indexes – insulin resistance, BMI; body mass index, ACE; angiotensin converting enzyme, A2; angiotensin II.

p-values refer to differences between the exercise and control group.

^a^Advanced vascular disease is defined as those with previous MI and/or diabetic microvascular complications in addition to coronary artery disease

Numbers of successfully analysed samples for gene expression in AT ranged from 41 to 82 at baseline, whereas the number after intervention ranged from 25 to 67, with somewhat unequal distribution between the intervention and control groups. Numbers are given in the result tables. The reduced number of AT samples was due to inadequate quantity and quality of samples and, in some instances, patients’ unwillingness to give fat tissue samples, particularly after the intervention period.

There were no significant between-group differences in changes in the weight, waist circumference and energy intake percentages of main nutrients or diabetes medication during the study period, as previously described[29].

Effects of exercise training

Levels of adipokines and TNFα before and after intervention are shown in Table 2 for the compliant population (n = 112). There was no statistically significant difference in changes during the intervention between the randomized groups in AT expression of adiponectin, visfatin and TNF. We did, however, observe a significantly increased visfatin expression in the exercise group (p = 0.007) and a reduction in adiponectin expression in the control group (p = 0.049).

**Table 2.** Levels of the measured markers at baseline and after 12 months in the randomized groups

	Control (n = 51)			Exercise (n = 61)				
	Baseline *(n)*	12 months *(n)*	p^1^	Baseline *(n)*	12 months *(n)*	P^2^	Δp	relΔp
Adiponectin – AT (RQ)	0.49 (0.39, 0.70) *(43)*	0.45 (0.240, 0.55) *(32)*	**0.049**	0.47 (0.37, 0.64) *(36)*	0.32 (0.17, 0.66) *(35)*	0.113	0.906	0.880
Visfatin – AT (RQ)	0.85 (0.62, 1.19) (*48)*	1.39 (0.82, 2.70) *(34)*	0.064	0.81 (0.65, 1.02) *(34)*	1.09 (0.74, 2.64) *(30)*	**0.007**	0.540	0.606
TNF – AT (RQ)	1.12 (0.64, 1.81) (*25)*	0.85 (0.44, 3.43) *(15)*	0.508	1.26 (0.95, 3.05) *(16)*	0.85 (0.53, 2.06) *(10)*	0.893	1.000	0.953
sAdiponectin (ug/ml)	1978 (1382, 3040) *(61)*	2121 (1205, 3318) *(61)*	0.719	2374 (1485, 3964) *(51)*	2525 (1728, 4355) *(51)*	0.216	0.334	0.305
sVisfatin (ug/ml)	1.78 (1.53, 2.14) *(61)*	1.82 (1.58, 2.11) *(61)*	0.232	1.72 (1.56, 2.10) *(51)*	1.93 (1.63, 2.23) *(51)*	**0.020**	0.460	0.435
sTNF (ug/ml)	0.89 (0.74, 1.04) *(61)*	0.87 (0.72, 1,12) *(61)*	0.152	0.83 (0.73, 0.97) *(51)*	0.96 (0.76, 1.04) *(51)*	**0.004**	0.220	0.172

Values are median (25, 75 percentile)

p^1^-values refer to changes within the control group from baseline to 12 months (Wilcoxon Signed Rank test)

p^2^-values refer to changes within the exercise group from baseline to 12 months (Wilcoxon Signed Rank test)

Delta p (Δ p) refers to the difference in the change between the groups from baseline to 12 months (Mann-Whitney test)

Relative delta p (relΔp) refers to the difference in the change between the groups from baseline to 12 months as related to the baseline values (Mann-Whitney)

AT; genetically expressed in adipose tissue, S; serum, RQ; Relatively Quantified

Bold text indicates statistically significant changes

There were also no statistically significant differences in changes between the groups in the circulating levels of neither adiponectin, visfatin nor TNF (Table 2). However, circulating levels of visfatin and TNF increased significantly in the exercise group (p = 0.020 and p = 0.004, respectively).

### Correlations at baseline

Table 3 shows baseline correlations between AT expression and circulating levels of the investigated markers, glucometabolic variables and VO_2_ peak in the total population (n = 137). AT expression of adiponectin was inversely correlated with BMI and positively correlated with the VO_2_ peak (r = −0.231 and r = 0.200, p < 0.05 for both). Circulating adiponectin correlated inversely with insulin, C-peptide and HOMA2-IR (p < 0.05 for all), but not with BMI or VO_2_ peak. Circulating TNF also correlated with insulin, C-peptide and HOMA2-IR (p < 0.05 for all), with an additional inverse correlation with the VO_2_ peak (r = −0.329, p < 0.001). After Bonferroni correction (48 comparisons), the correlations between circulating adiponectin and insulin (r = −0.271) and between circulating TNF levels and C-peptide and VO_2_ peak remained statistically significant (r = −0.278 and r = −0.329, respectively, p ≤ 0.001 for all).

**Table 3.** Baseline correlations of the measured biomarkers and glucometabolic variables and VO_2_ peak in the total population (n = 137)

	Years with diabetes	Glucose	HbA1c	Insulin	c-peptide	HOMA2-IR	BMI	VO_2_peak
Adiponectin – AT *(n = 101)*	r = 0.012 p = 0.907	r = −0.077 p = 0.449	r = −0.116 p = 0.247	r = −0.169 p = 0.091	r = −0.154 p = 0.123	r = −0.166 p = 0.109	**r = −0.231** **p = 0.020**	**r = 0.200** **p = 0.045**
Visfatin – AT *(n = 99)*	r = 0.042 p = 0.688	r = −0.069 p = 0.502	r = −0.112 p = 0.268	r = −0.117 p = 0.250	r = −0.016 p = 0.877	r = −0.111 p = 0.294	r = −0.039 p = 0.699	r = −0.008 p = 0.954
TNF – AT *(n = 48)*	r = −0.074 p = 0.623	r = −0.121 p = 0.423	r = −0.143 p = 0.332	r = −0.111 p = 0.452	r = −0.123 p = 0.406	r = −0.072 p = 0.638	r = −0.195 p = 0.185	r = 0.111 p = 0.274
sAdiponectin *(n = 137)*	r = 0.075 p = 0.387	r = −0.018 p = 0.840	r = −0.029 p = 0.740	**r = −0.271** p = 0.001*	**r = −0.179** **p = 0.037**	**r = −0.242** **p = 0.006**	r = −0.006 p = 0.942	r = −0.156 p = 0.069
sTNF *(n = 137)*	r = 0.134 p = 0.123	r = 0.036 p = 0.682	r = 0.086 p = 0.320	**r = 0.178** **p = 0.037**	**r = −0.278** p = 0.001*	**r = 0.190** **p = 0.033**	r = 0.156 p = 0.070	**r = −0.329** p < 0.001*
sVisfatin *(n = 137)*	r = −0.121 p = 0.164	r = −0.150 p = 0.083	r = −0.050 p = 0.560	r = 0.080 p = 0.354	r = 0.058 p = 0.499	r = 0.069 p = 0.446	r = −0.009 p = 0.917	r = 0.063 p = 0.464

AT; adipose tissue, s; Serum

Bold text indicates a statistically significant correlation

*significant after Bonferroni correction (p = 0.001 by 48 performed associations)

Associations between serum levels and gene expression of adipokines at baseline and after intervention

We investigated the correlations between serum levels and gene expression of the investigated adipokines at baseline and after 12 months. We found an inverse correlation between circulating visfatin and gene expression of TNF in the control group (r = −0.401, p = 0.047) and between circulating visfatin and visfatin gene expression in the exercise group (r = −0.359, p = 0.037) (Supplementary Table 1) at baseline. We also found an inverse correlation between circulating and genetically expressed TNF in the exercise group, after intervention (r = −0.636, p = 0.048) (Supplementary Table 2). The observed associations were no longer statistically significant after Bonferroni correction (18 comparisons).

Associations between changes in adipokines and the VO_2_ peak

When investigating delta values (i.e. changes from baseline to 12 months) for adiponectin, visfatin and TNF and VO_2_ peak after 1 year, we observed a significant correlation between delta circulating adiponectin levels and the delta VO_2_ peak in the compliant population (n = 112) (r = 0.256, p = 0.008) as shown in Table 4.

**Table 4.** Correlations between the change (Δ) in the measured variables and the change in the VO_2_ peak (n = 112)

	ΔVO_2_peak
ΔAdiponectin – AT *n = 55*	r = 0.072 p = 0.612
ΔVisfatin – AT *n = 50*	r = 0.156 p = 0.279
ΔTNFα – AT *n = 15*	r = 0.345 p = 0.227
ΔsAdiponectin *n = 112*	**r = 0.256** **p = 0.008**
ΔsVisfatin *n = 112*	r = 0.074 p = 0.450
ΔsTNFα *n = 112*	r = 0.008 p = 0.935

AT; adipose tissue; s; Serum

Bold text indicates statistically significant correlation

## Discussion

The main finding in our study was that long-term exercise intervention did not affect AT expression and circulating levels of adiponectin, visfatin and TNF significantly. We could, however, show an association between the improved VO_2_ peak and increased serum adiponectin levels.

Previous studies examining the exercise effect on adiponectin suggest that a prerequisite to change in adiponectin levels, both genetically expressed in AT and circulating levels, is a 10–15% weight loss[31]. In our population, there was no significant change in the weight before and after the intervention, which in part may explain the absence of significant changes in adipokines. Many patients were unable to follow the exercise program for a longer and shorter period with a relatively high prevalence of somatic symptoms and complaints, such as angina, affecting the compliance to the exercise intervention[29]. Furthermore, there were no dietary interventions in either groups[29]. Finally, patients who were already engaged in an exercise program were accepted into the study, as long as their level of activity did not increase from the time of inclusion, which could have resulted in higher baseline fitness and lower potential for further weight loss. This could explain the absence of significant weight loss in our study. Inconsistent findings exist in this regard, as some studies do suggest that exercise intervention regardless of weight loss might increase circulating adiponectin levels[32]. Saunders et al. showed that aerobic exercise training significantly increased plasma adiponectin without changes in the weight or waist circumference[33]. This study was, however, performed in a younger group of participants without concomitant disease and the plasma adiponectin levels were measured shortly after an acute bout of exercise.

A study performed on abdominally obese men showed an elevation in circulating adiponectin levels immediately after an acute bout of exercise compared to baseline, which remained elevated for at least 30 min of rest[33]. One would have to question whether daily fluctuations and variation in circulating adiponectin levels may play a role. In our study, blood samples were retrieved within one week after the last exercise bout[29].

TNF increased significantly in the exercise group and non-significantly in the control group, so the difference in change was not significant. This could possibly be because in patients with longstanding CAD and T2DM, the AT may develop into a more pro-inflammatory state, potentially rendering rigorous exercise training in such patients non-beneficial. Although previous findings mainly suggest otherwise [34], the populations in these studies had not yet developed complications from their T2DM. It might thus be suggested that the anti-inflammatory effects of exercise are primarily seen preventively, before developing complications. Our population had already developed CAD.

When exploring baseline associations, we found that AT expression of adiponectin was inversely correlated with BMI and positively correlated with the VO_2_ peak. This is consistent with previous findings[35]. It is discussed that obesity per se contributes to an alteration in the expression of adiponectin and decreases its receptors’ susceptibility, leading to insulin resistance and its consequences[36]. In a study by Bjornstad et al, both low circumferential strain on echocardiography and low VO_2_ peak were shown to be independently related to low levels of circulating adiponectin[35]. This is in congruence with the anti-atherogenic properties of adiponectin, indicating its cardioprotective role against CVD and thereby a potential physiological explanation for the associations reported between circulating adiponectin and VO_2_peak[37]. Animal models have also shown that adiponectin-deficient mice exhibited an exacerbated myocardial contractile dysfunction with increased interstitial fibrosis compared to wild-type mice, 4 weeks after creation of MI through left anterior descending artery ligation[38]. A study performed on 528 healthy Japanese participants revealed a correlation between the VO_2_ peak and circulating adiponectin, which, however, attenuated when adjusted for BMI. This suggests that BMI may be a confounder in these associations, which may further imply that the quantity of AT is the decisive factor for circulating levels of adiponectin[39]. In our study, we could also demonstrate a positive correlation between the change in the VO_2_ peak and the change in circulating adiponectin, which is in line with the aforementioned findings.

At baseline, we found an inverse correlation between circulating adiponectin and insulin, C-peptide and HOMA2-IR. However, after Bonferroni correction, only the inverse correlation between insulin and adiponectin remained significant. Although previous reports have mainly focused on the relationship between insulin resistance/insulin sensitivity and adiponectin, the results are somewhat in accordance with these findings, suggesting a protective role of adiponectin against insulin resistance and MetS [40,41]. There are also studies suggesting that low levels of adiponectin in people with T2DM and obesity are mostly attributable to insulin resistance and hyperinsulinemia[42].

When investigating the associations between circulating levels and gene expression of adipokines, we found an inverse correlation between circulating and AT-expressed visfatin and between circulating visfatin and TNF gene expression at baseline. After the intervention, we found an inverse correlation between circulating and genetically expressed TNF in the exercise group. However, none remained significant after bonferroni correction. This could be indicative of a downregulation at the gene expression level with increased circulating visfatin and TNF. Nonetheless, one has to consider the limited amount of representative AT samples, particularly for TNF. Furthermore, other potential sources for adipokines, such as deeper layers of the AT, have to be considered.

AT expression and circulating levels of visfatin and circulating TNF increased, surprisingly in the exercise group, but the differences in changes from controls were not significant. Considering their potential deleterious effect on glucometabolism and cardiovascular system, we anticipated a reduction in TNFα and visfatin after exercise training; however, the majority of previous studies, although performed in different populations, have shown that circulating TNFα remains unchanged following exercise [43–45]. Ryan et al. showed that weight loss induced by diet and exercise resulted in a significant reduction in fasting glucose and increased insulin sensitivity, but no changes in TNFα serum concentrations[46]. One might, therefore, question whether the local response of TNFα is more important in the glucose metabolism than the systemic response.

Nevertheless, we found that TNFα correlated inversely with C-peptide and teh VO_2_ peak at baseline, which is significant after Bonferroni correction. In line with this, one study has shown significant improvement in the VO_2_ peak 4 months after treatment with anti-TNFα therapy[47].

Levels of visfatin have been shown to decrease in obese, non-diabetic and diabetic subjects, following 12 weeks of both aerobic and resistance exercise training, and the reduction seems to be related to changes in both glucose and insulin, but not necessarily to changes in weight [48–51]. The absence of any relationship between visfatin, exercise training and VO_2_ peak in our study may be multifactorial. Our population with many years of T2DM had developed vascular complications and were heavily medicated on ACE inhibitors, statins, platelet inhibitors and anti-diabetic medication that might have affected the outcome, which maybe due to some degree of irreversible changes in the AT composition. Furthermore, previous reviews on the effects of exercise on adipokines such as visfatin suggest that reduction in central fat percentage and change in AT composition are a necessity to observe changes in circulating levels of adipokines[27].

## Limitations

The main limitation of our study is that it was designed to study the effect of exercise training on HbA1c [29], thus not to answer our hypothesis. However, performing power calculations for studies on biomarkers not ordinarily applied in the clinical setting is a challenge in itself. The reluctance of some participants to give AT samples also limited the number of AT samples available.

The absence of weight reduction is unfortunate. As mentioned, several studies suggest that loss of weight (through both diet and exercise) seems to be the main driving factor towards desired effects on adipokines, which is plausible, considering that AT is the main source. Whether the amount of exercise in our population was sufficient could also be discussed. Furthermore, any physical activity in the control group was not accounted for, which might have influenced the results of our study.

Adiponectin circulates in blood in many isoforms and the high-molecular weight adiponectin is thought to be most biologically active in the uptake of glucose and insulin sensitivity[52]. We have not differentiated between the isoforms, which is also a limitation in our study. However, measuring isoforms is challenging, particularly because there is a lack of a gold standard[52].

Our study population was heavily medicated with ACE inhibitors/A2-blockers, statins and antidiabetics. Some of these medications have anti-inflammatory properties, such as statins and metformin, which might have affected the circulating levels of adiponectin and visfatin and thus masked the potential beneficial effect of exercise training [53,54].

The strength of our study was the randomization principle applied with equal distribution between both groups. The number of participants in our study exceeds numerous studies that have addressed the same issue.

## Conclusion

In our combined T2DM and CAD population, exercise training without weight loss had a limited impact on AT expression and circulating levels of adipokines. Adiponectin was associated with the VO_2_ peak, suggesting a potential association with cardiorespiratory fitness.

## Supplementary Material

Supplemental Material

## Data Availability

There are no publicly available data sets or code due to national ethical restrictions in protection of human subjects. The data will be shared in accordance with local registration and ethical approval on reasonable request to the corresponding author
