# Supplementary material for: Adiponectin in relation to exercise and physical performance in patients with type 2 diabetes and coronary artery disease
Source: Adipocyte. 2021 Nov 14;10(1):612–20. doi: 10.1080/21623945.2021.1996699 (PMC8726619; doi:10.1080/21623945.2021.1996699)
Supplement: Supplemental Material [file KADI_A_1996699_SM4287.docx]

**Supplementary Table 1 – Correlation between circulating levels and gene expression of adipokines at baseline in both randomized groups**

|  | Control (n=51) | | |  | Exercise (n=61) | | |
| --- | --- | --- | --- | --- | --- | --- | --- |
|  | AT-Adiponectin | AT-Visfatin | AT-TNF |  | AT-Adiponectin | AT-Visfatin | AT-TNF |
| sAdiponectin | r=0.224  p=0.154 | r=-0.068  p=0.648 | r=-0.014  p=0.948 |  | r=0.016  p=0.928 | r=0.307  p=0.078 | r=-0.294  p=0.269 |
| sVisfatin | r=0.006  p=0.970 | r=-0.069  p=0.643 | **r=-0.401**  **p=0.047** |  | r=-0.420  p=0.810 | **r=-0.359**  **p=0.037** | r=0.021  p=0.940 |
| sTNF | r=-0.243  p=0.122 | r=-0.026  p=0.859 | r=0.253  p=0.222 |  | r=-0.258  p=0.129 | r=-0.025  p=0.888 | r=-0.003  p=0.991 |

s; serum

AT; adipose tissue (gene expression in adipose tissue)

Bold text indicates p-value <0.05

Bonferroni correction (p = 0.003 by 18 performed associations)

**Supplementary Table 2** **– Correlation between circulating levels and gene expression of adipokines after intervention in both randomized groups**

|  | Control (n=51) | | |  | Exercise (n=61) | | |
| --- | --- | --- | --- | --- | --- | --- | --- |
|  | AT-Adiponectin | AT-Visfatin | AT-TNF |  | AT-Adiponectin | AT-Visfatin | AT-TNF |
| sAdiponectin | r=0.039  p=0.833 | r=-0.119  p=0.503 | r=-0.321  p=0.243 |  | r=0.161  p=0.355 | r=-0.095  p=0.619 | r=0.115  p=0.751 |
| sVisfatin | r=-0.050  p=0.786 | r=-0.159  p=0.370 | r=-0.264  p=0.341 |  | r=-0.097  p=0.581 | r=0.213  p=0.259 | r=-0.200  p=0.580 |
| sTNF | r=-0.116  p=0.527 | r=0.150  p=0.398 | r=0.089  p=0.752 |  | r=-0.245  p=0.155 | r=-0.109  p=0.567 | **r=-0.636**  **p=0.048** |

s; serum

AT; adipose tissue (gene expression in adipose tissue)

Bold text indicates p-value <0.05

Bonferroni correction (p = 0.003 by 18 performed associations)
